# Bioluminescent imaging of vaccinia virus infection in immunocompetent and immunodeficient rats as a model for human smallpox

**DOI:** 10.1038/srep11397

**Published:** 2015-08-03

**Authors:** Qiang Liu, Changfa Fan, Shuya Zhou, Yanan Guo, Qin Zuo, Jian Ma, Susu Liu, Xi Wu, Zexu Peng, Tao Fan, Chaoshe Guo, Yuelei Shen, Weijin Huang, Baowen Li, Zhengming He, Youchun Wang

**Affiliations:** 1 Division of HIV/AIDS and Sex-transmitted Virus Vaccines, Key Laboratory of the Ministry of Health for Research on Quality and Standardization of Biotech Products, National Institutes for Food and Drug Control, Beijing, 100050, China; 2 Division of Animal Model Research, Institute for Laboratory Animal Resources, National Institutes for Food and Drug Control, Beijing, 100050, China; 3 Biocytogen Co., Ltd, Beijing, 101111, China

## Abstract

Due to the increasing concern of using smallpox virus as biological weapons for terrorist attack, there is renewed interest in studying the pathogenesis of human smallpox and development of new therapies. Animal models are highly demanded for efficacy and safety examination of new vaccines and therapeutic drugs. Here, we demonstrated that both wild type and immunodeficient rats infected with an engineered vaccinia virus carrying Firefly luciferase reporter gene (rTV-Fluc) could recapitulate infectious and clinical features of human smallpox. Vaccinia viral infection in wild type Sprague-Dawley (SD) rats displayed a diffusible pattern in various organs, including liver, head and limbs. The intensity of bioluminescence generated from rTV-Fluc correlated well with viral loads in tissues. Moreover, neutralizing antibodies had a protective effect against virus reinfection. The recombination activating gene 2 (Rag2) knockout rats generated by transcription activator-like effector nucleases (TALENs) technology were further used to examine the infectivity of the rTV-Fluc in immunodeficient populations. Here we demonstrated that Rag2-/- rats were more susceptible to rTV-Fluc than SD rats with a slower virus clearance rate. Therefore, the rTV-Fluc/SD rats and rTV-Fluc/Rag2-/- rats are suitable visualization models, which recapitulate wild type or immunodeficient populations respectively, for testing human smallpox vaccine and antiviral drugs.

Smallpox, caused by variola virus (VARV), is one of the most devastating diseases to humans, and has been eradicated in 1980^1^. Currently, VARV is legally held in two high-security laboratories: CDC in Atlanta USA and VECTOR in Koltsovo Russia^2^. However, VARV continues to be discovered outside these two WHO-sanctioned laboratories, e.g. Eastern Europe in the 1990s and more recently in USA^3^. Notably, the cessation of vaccination for over 30 years results in immunologically naive human population that would be at high risk if VARV is used as an agent of bioterrorism^4^. A better understanding of smallpox virus infection, and development of antiviral agents and novel vaccines would mitigate this threat^5^.

In recent years, vaccine efficacy cannot be examined directly against smallpox, because smallpox virus no longer occurred in human population. The efficacy of candidate vaccines can only be predicted in animals infected with orthopoxvirus, such as mouse/vaccinia virus^6^, mouse/mousepox^7^, rabbit/rabbitpox^8^ and monkey/monkeypox^9^. Due to many advantages of rats, including short life span, easy to be manipulated, and similar pathological/physiology features to humans for major health issues^10^, rats are better animal models than mice for studying human diseases. Once rat genome sequence became available, the frequency of using genetically modified rats in biomedical research is greatly increased these years.

It has been speculated that the immunodeficient individuals would be more susceptible to smallpox and have a greater risk of severe morbidity and mortality after smallpox virus infection^11^. Although there is scarce evidence directly supporting this claim, it is clear that inadvertent inoculation of smallpox vaccine result in severe complications and death in children with T-cell deficiency, or in the immunocompromised population caused by AIDS or immune suppression drugs^12^,^13^. Therefore, we generated immunodeficient Rag2-/- rat to resemble the immunodeficient human populations and examine the orthopoxvirus infection course.

Vaccinia virus (VACV) belongs to the orthopoxvirus (OPV) and represents a valuable surrogate virus^14^,^15^. Previously, we investigated the replication characteristics of replication-competent vaccinia tiantan strain in mice using an *in vivo* bioluminescent imaging (BLI) method with a recombinant vaccinia expressing firefly luciferase (rTV-Fluc)^16^,^17^. The BLI technology can monitor pathogen dissemination in real time, and locate pathogens residing in unexpected anatomical sites^18^,^19^. The aim of the present study was to real-time monitoring the vaccinia viral infection in living rats with or without immunodeficiency.

Here we reported that the pathogenesis of VACV infection course, including the initial local replication and systemic dissemination following viremia, closely resembled human infection with smallpox. Furthermore, we revealed that the neutralizing antibody production could be stimulated by VACV infection, and the antibody titers correlated well with the protective effectiveness from the rTV-Fluc reinfection. These results indicated that the usage of rats infected with VACV could be used as a model to evaluate OPV candidate vaccines. We also demonstrated that the immunodeficient Rag2-/- rats with deficiency of T/B cells were more susceptible to rTV-Fluc infection, with extended infection duration, less weight gain, and pathological changes in the spleen, kidney and lung. Therefore, rTV-Fluc/Rag2-/- rats can serve as a faithful model of immunodeficient population infected with human smallpox to evaluate the safety and efficacy of OPV antiviral drugs.

## Results

### Generation and characterization of Rag2-/- rats

**Figure 1:**
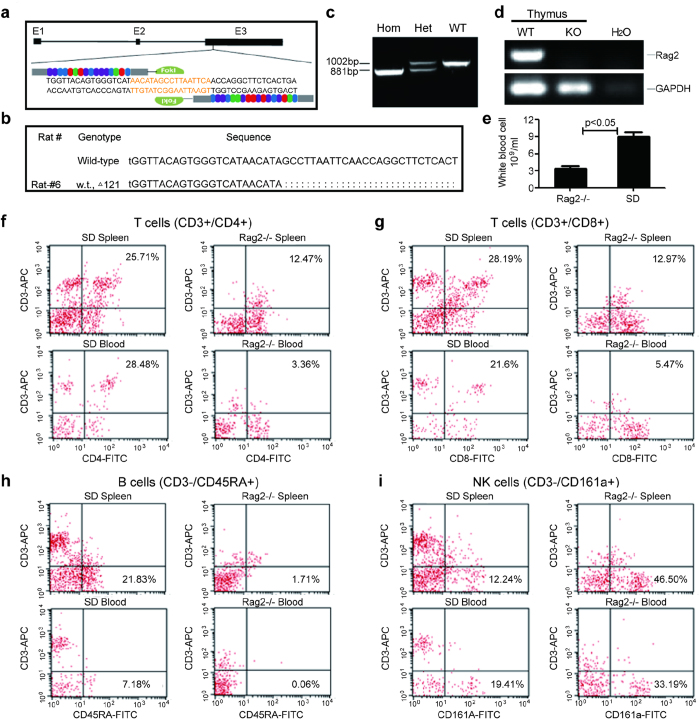
Generation and characterization of Rag2-/- rats. (**a**) Schematic representation of Rag2 gene deletion targeting strategy. Exon 3 of Rag2 gene was targeted with the TALEN genome editing technology. (**b**) Sequencing of the TALEN targeting site identified a 121bp deletion resulting in a frame shift of the Rag2 gene. (**c**) Genotyping analysis of Rag2-/- heterozygous and homozygous rats using specific primers to amplify Rag2 gene from the tail DNA. WT band, 1002bp; knockout band 881bp. (**d**) Detection of Rag2 mRNA expression in the thymus by RT-PCR analysis. The Rag2 primer amplifed a 461bp band only in wildtype rat thymus, while GAPDH primers were used as controls and amplifed a band in both wildtype and Rag2-/- rats. (**e**) Analysis of peripheral white blood cell counts in Rag2-/- and SD rats (n = 10). (**f**–**i**) The presence of various lymphocyte populations was examined by flow cytometry in the peripheral blood and spleen of SD and Rag2-/- rat. The T cell compartment was analyzed by simultaneously staining either with APC-CD3 and FITC-CD4 (**f**) or APC-CD3 and FITC-CD8 (**g**). To analyze B cell compartment, cells were stained with APC-CD3 and FITC-CD45RA (**h**). The NK cells were revealed with double staining of APC-CD3 and FITC-CD161a (**i**). The percentages of cells positive for a given phenotype are indicated by the numbers.

Rag2 gene encodes a protein that is involved in the initiation of V(D)J recombination during B and T cell development. To delete the Rag2 gene from the genome to generate an immunodeficient rat, we decided to target exon 3 of Rag2 gene of Sprague-Dawley (SD) rat using the TALEN genome editing technology (Fig. 1a). The TALEN repeats were fused with FokI restriction endonuclease and transfected into the C6 cell line. The expression and cleavage activity of TALEN proteins were verified through western blot and MSDase assay, respectively. TALEN mRNA was then *in vitro* transcribed and microinjected into the cytoplasm of SD rat zygotes (n = 456). Six Rag2-/- rats were generated after transferring 338 embryos into 16 surrogate female rats. The highest Rag2 mutation frequency was derived from an intracytoplasmic injection dose of 20 ng/μl. Further sequencing of the TALEN targeting site showed mutations in the Rag2 gene: a 121 bp deletion resulting in a frame shift (Fig. 1b). From F0 founder rats, we further generated Rag2 deficient heterozygous and homozygous rats determined by genotyping (Fig. 1c). The loss of Rag2 mRNA expression also indicated successfully knocking out of Rag2 gene (Fig. 1d). We observed that the Rag2-/- rats were viable, fertile and did not show noticeable physical abnormalities. Compared to immunocompetent SD rats, the white blood cell numbers in Rag2-/- rats were significantly reduced, suggesting that the knocking out of Rag2 gene affects the development and maturation of white blood cells (P < 0.05, Fig. 1e).

To further examine the T and B cell development in homozygous Rag2 knockout rat, we performed flow cytometry to identify various white blood cells in the peripheral blood and spleen. T lymphocyte differentiation generally proceeds from null cells to CD4^+^/CD8^+^ cells and then to cells that express either CD4 or CD8 alone. The subset T helper cells are CD3^+^/CD4^+^ while the subset T cytotoxic cells are CD3^+^/CD8^+^. We detected significantly reduced number of T helper cells (CD3^+^/CD4^+^) and T cytotoxic cells (CD3^+^/CD8^+^) in the spleen of Rag2-/- compared with wild type rat. This blockage of T cell development was further confirmed by almost undetectable T helper cells (3.36%) and T cytotoxic cells (5.47%) in the peripheral blood of Rag2-/- rat (Fig. 1f,g). In addition, we failed to detect CD3^-^/CD45RA^+^ B cells in both spleen cells (1.71%) and the peripheral blood (0.06%) of Rag 2-/- rats (Fig. 1h). These results indicated that there were no mature T and B cells after Rag2 gene was knocked out. However, we observed that the number of CD3^-^/CD161a^+^ NK cells (Fig. 1i) was increased in Rag2-/- rats (spleen cells 46.50% and peripheral blood 33.19%) compared with wild type rats (spleen cells 12.24% and peripheral blood 19.41%). This NK cell increase may reflect a compensation mechanism to strength the immune system due to the lack of mature T and B cells caused by Rag2 gene knockout.

### Bioluminescent imaging of rTV-Fluc infection in living rats

**Figure 2:**
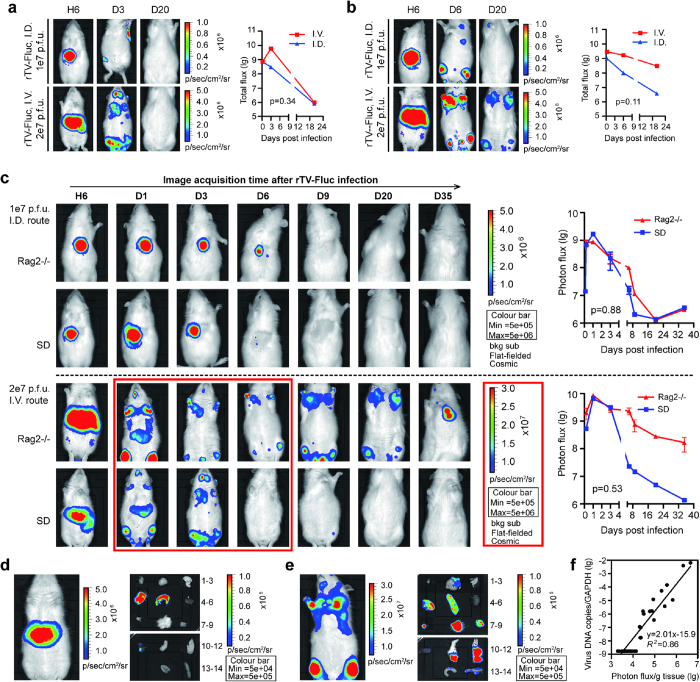
Bioluminescence imaging of immunocompetent SD and immunodeficient Rag2-/- rats infected with rTV-Fluc. The replication and dissemination of rTV-Fluc in SD rats (**a**) and Rag2-/- rats (**b**). Three 4-week old female SD and Rag2-/- rats were inoculated with rTV-Fluc by intradermal (1 × 10^7^ p.f.u) and intravenous injection (2 × 10^7^ pfu), respectively. The relative level of bioluminescence was shown in pseudocolor, with red and blue representing the strongest and weakest photon fluxes, respectively. Since the dorsal images couldn’t visualize the dissemination of virus to the limbs, ventral images were shown here except the initial time point of ID injection. Values of total flux for each groups was shown on the right and each data point represents a mean value (n = 3). (**c**) Comparison of the intensity and duration of bioluminescence between SD rats and Rag2-/- rats inoculated via ID or IV injection. Bioluminescent images were superimposed on gray-scale photographs of rats at 6h, 1d, 3d, 6d, 9d, 20d and 35d post infection. A representative animal from each group was shown. Each data point represents mean ± SEM (n = 3). The differences between two subgroups were calculated using paired t-test. The living SD rat intravenously infected with 2 × 10^7^ pfu rTV-Fluc was imaged at 6 hours (**d**) and 1 day (**e**) after infection. The different organs and tissues were dissected and imaged. 1) lymph node, 2) brain, 3) lung, 4) live; 5) spleen; 6) kidney; 7) ovary; 8) skin; 9) muscle; 10) heart; 11) fore limb; 12) hind limb; 13) large intestine; 14) small intestine. (**f**) Correlation of rTV-Fluc viral loads in various tissues to the intensity of bioluminescence at 1d and 35d post infection.

For rTV-Fluc/immunocompetent SD rat model, animals were infected with 1 × 10^7^ pfu of rTV-Fluc by intradermal (ID) and 2 × 10^7^ pfu via intravenous (IV) inoculation, respectively. Bioluminescent imaging (BLI) was performed on all animals to monitor the progression, sites and degree of viral infection based on the distribution and amounts of detected luciferase photons. *In vivo* luminescent signal could be visualized on the inoculation site of ID injection or in the abdomen via IV injection as early as 6 hours post infection, suggesting that bioluminescent signal generated from rTV-Luc is sensitive enough for detecting virus infection even in a very early stage. By comparison, infection via IV route produced a more intense signal than that via ID route, indicating that a higher proportion of IV inoculated virus infiltrated into the blood and spread quickly to other organs (Fig. 2a). By day 3, systemic dissemination in SD rats was observed from the ID inoculation site to limbs, or more quickly from IV inoculation site to heads and limbs (Fig. 2a). In both ID and IV inoculation groups, ventral luminescent signals in whole body and dissected tissues including brain, ovary, skin, limbs, liver, spleen, kidney, and lung were undetectable by day 20 (Supplementary Fig. 1), indicating that the virus had been cleared by that time. In contrast, in the rTV-Fluc/immunodeficient Rag2-/- rat model, luminescent signal was readily visible at 6 hours post infection and had a similar distribution pattern as that in rTV-Fluc/immunocompetent rats. In Rag2-/- rats, IV inoculation produced a more intense luminescent signal spreading to other organs in limb and head region by day 1 post infection. Furthermore, luminescent signal was still detectable in limbs at day 35 post infection, indicating that rTV-Fluc virus could stay longer in immunodeficient rats than in immunocompetent SD rats (Fig. 2c).

To assess the role of T and B cells on antiviral effects, we compared the bioluminescence between Rag2-deficient and immunocompetent SD rats. For the initial 3 days post infection, there was no difference in the bioluminescence intensity between two groups. The infection time course was significantly increased in Rag2-/- rats than that in SD rats after IV inoculation (>35 days IV vs <6 days IV, p < 0.05). The bioluminescence could be detected as early as 6 hours, peaked at 1 day and remained visible for more than 35 days post infection. However, after ID inoculation the duration and intensity of bioluminescence, observed from the back, were comparable between the Rag2-deficient and SD rats (p = 0.88, Fig. 2c), possibly because the antiviral effect was mainly relied on NK cells in the subcutaneous tissue. A repetitive experiment was performed and produced an almost identical result (Supplementary Fig. 1).

In terms of the bioluminescence dissemination *in vivo*, different organs or tissues from two groups of IV infected rats were imaged after perfusion of the luciferase substrate luciferin. The bioluminescence could be detected in liver and spleen at 6 hours (Fig. 2d), and more organs including brain, ovary, skin and limbs at day 1 post infection (Fig. 2e). These results reflected the dissemination kinetics and suggested that the rTV-Fluc virus in the abdomen and limbs were spread from the liver or spleen. Notably, by *ex vivo* imaging of infected Rag2-/- rat brain, we found that the luminescence was restricted to a focal area in the posterior part of the brain, possibly the cerebellum and brain stems. In the meanwhile, different tissues were sampled to examine the correlation between the luminescence intensity and viral loads. The rTV-Fluc viral loads in tissues were measured by quantitative PCR and presented as virus DNA copies/GAPDH ratios. A strong correlation between bioluminescence intensity and viral loads was observed (R^2^ = 0.86), proving that the luminescent intensity can be used as an index for rTV-Fluc viral infection. Based on the calibration curve, approximate viral DNA load could be calculated using the following formula: lg viral load = 2.01 lg photo flux/g tissue-15.9 (Fig. 2f).

### Pathogenicity of rTV-Fluc in Rag2-/- and SD rats

**Figure 3:**
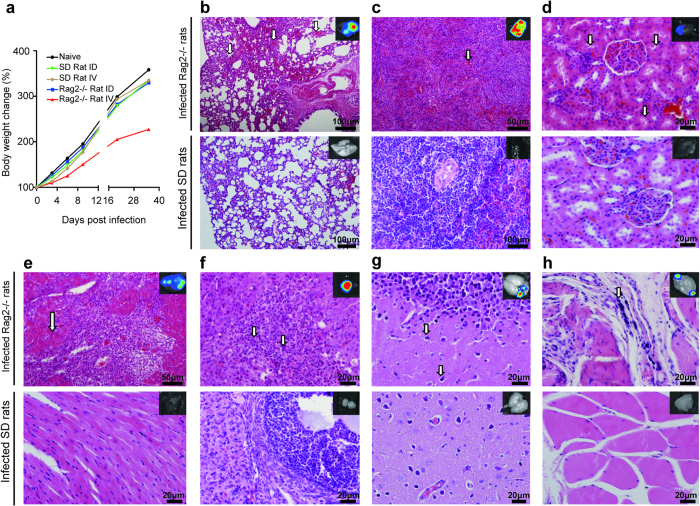
The rTV-Fluc infection of Rag2-/- rats resulted in less weight gain and pathological tissue injuries. (**a**) The body weights of SD and Rag2-/- rats infected with rTV-Fluc via ID or IV routes were recorded (n = 3). Histopathological analysis of lung (**b**), spleen (**c**), kidney (**d**), heart (**e**), ovary (**f**), brain (**g**), and muscle (**h**) at day 35 post infection of IV 2 × 10^7^ pfu of rTV-Fluc. Paraffin fixed tissue sections were stained with haematoxylin and eosin, and arrows indicated lesion sites. Scale bar, 20–100 μm. The bioluminescent imaging of each organ is displayed on the upper right, with colors ranging from 1e^4^ p/sec/cm^2^/sr to 1e^5^ p/sec/cm^2^/sr.

The vaccinia virus tiantan strain is a highly attenuated vaccine strain, which has been passaged serially in several species including human, monkey and cattle, etc. To analyze whether rTV-Fluc has pathogenicity, Rag2-/- and SD rats were infected with rTV-Fluc by either ID or IV inoculation. The body weight gain in IV infected Rag2-/- rats was significantly less than in other groups after infection (Fig. 3a). No death were observed in both immunocompetent SD and immunodeficient Rag2-/- rats after infection, and systemic histopathology examination was performed for many tissues including brain stem, cerebellum, mouth, heart, liver, spleen, lung, kidney, intestine, testis and ovary. Rag2 deficient rats infected with rTV-Fluc displayed evidences of hemorrhage in the lung (Fig. 3b, red blood cell filled in the pulmonary alveoli, indicated by arrows), spleen (Fig. 3c) and kidney (Fig. 3d, red blood cells were observed in renal vascular). Kidney epithelial cells loss and albumin proteinosis occurred occasionally in the nephric tubules (Fig. 3d, indicated by arrows). Massive hyperplasia of connective tissues was seen in the lung and heart (Fig. 3b,e). In addition, inflammatory cells were infiltrated into the heart, lung and ovary. A huge necrotic area was also observed in the ovary (Fig. 3f, indicated by arrows), which might be a cardinal symptom related to infection. Abnormal glial cells and lymphoid cells were also observed in the brain and muscle (Fig. 3g,h, indicated by arrows). However, no pathological injuries were identified in any organ of SD rats after IV infection at 35 days. In conclusion, there was a strong relationship between viral infection and pathological tissue injuries in immunodeficient Rag2-/- rats.

### The protective effect of neutralizing antibody against rTV-Fluc reinfection

**Figure 4:**
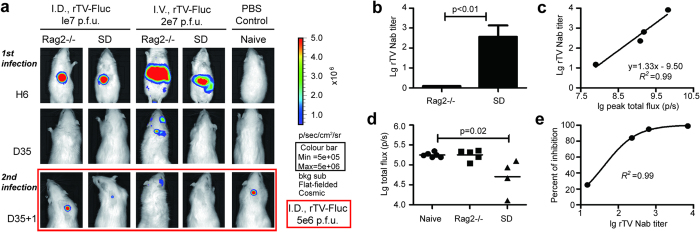
Evaluation of the antiviral efficacy of the neutralizing antibodies *in vivo* using the bioluminescence imaging of rTV-Fluc. (**a**) Bioluminescence imaging of Rag2-/- and SD rats. At 35d post initial inoculation, rats infected with rTV-Fluc via ID or IV inoculation were reinfected with 5 × 10^6^ p.f.u rTV-Fluc by ID injection, and naive rats were inoculated with PBS as controls. A representative animal from each group was shown. (**b**) The vaccinia virus neutralizing antibody titers were determined in Rag2-/- and SD rats at 35d post initial infection. Each data column represents mean ± SEM (n = 6). (**c**) Correlation of total flux peak in SD rats at 1d post initial infection to neutralizing antibody titers detected at 35 days later. (**d**) Values of total flux for Rag2-/- and SD rats at 1d after reinfection, and naive SD rats as controls. The statistical significance was calculated using ANOVA. (**e**) Correlation of the neutralizing antibody titers with the inhibition percentage of bioluminescent intensity at day 1 post reinfection.

To analyze whether the rTV-Fluc/rats models could be used to evaluate the antiviral effects of vaccines *in vivo*, at day 35 post initial infection rats were sampled to examine VACV-specific antibody titers by an *in vitro* chemiluminescence-based neutralizing antibody test. On day 36, rats were reinfected with 5 × 10^6^ pfu rTV-Fluc by ID injection (Fig. 4a). No neutralizing antibodies were generated and detected in Rag2-/- rats, possibly due to the lack of mature T and B cells. However, neutralizing antibody with various titers were detected in immunocompetent SD rats at day 35 (Fig. 4b), and the antibody titers has a strong correlated with the peak intensity of bioluminescence at day 1 (Fig. 4c). We also found that there was no difference between the bioluminescence intensity in Rag2-/- and naive rats (p = 0.91) after reinfection at day 36, indicating that no immune response was induced by rTV-Fluc initial infection in Rag2-/- rats. However, the intensity of bioluminescence of rTV-Fluc was much lower in SD rats (p = 0.02, Fig. 4d), suggesting that the neutralizing antibody generated in immunocompetent rats could defend viral reinfection. Moreover, the percentage of bioluminescence inhibition was correlated with the neutralizing antibodies tiers in SD rats (R^2^ = 0.99, Fig. 4e), further proved the antiviral protecting effect of neutralizing antibodies. Overall, the rTV-Fluc/rats are convenient and valuable tools for investigating virus replication and antiviral therapeutics in living rats.

## Discussion

In this study, we for the first time demonstrate a valuable tool to visualize the infection of VACV in living rats with and without immunodeficiency. This is a significant advance toward the development of antiviral therapeutics and vaccine improvement, as well as the understanding of variola pathogenesis. The ‘Animal Rule’ issued by US FDA states that efficacy data can be obtained from appropriate animal models and bridged to humans. Because of the small size, easy manipulation and breeding characteristics, laboratory rat is a preferred model system for many biomedical studies, such as physiology, pharmacology, toxicology, immunology and many diseases. The utilization of genetically modified rats as animal models is highly restricted by limited gene targeting technologies that can be applied to rats. Since new gene targeting technologies have been developed in the recent years, genetically modified rat models start to be widely used since 2009^20^,^21^,^22^. Compared with other genome editing technologies, such as ZFNs and CRISPR/Cas9 system, TALENs offer several advantages, including the ability to bind any sequence in the genome, high specificity and low cytotoxicity^23^. In this study, the Rag2 knockout rats were generated with TALEN technology, and among the firsts to be used to study VACV infection and the antiviral mechanism with an *in vivo* bioluminescence imaging.

BLI technique has been used to study various bacterial and viral pathogens by detecting luminescent marked targets (pathogens and cells) in live animals. We and others also demonstrate the advantages of using BLI to investigate the progression, replication and localization of viral infection in intact animals. More importantly BLI can identify unexpected infection sites and patterns that could be easily missed without real time imaging^17^,^24^, and it helps to predict the lethality of viral infected mice^19^. The good correlation between the bioluminescence intensity with viral loads in various tissues identified in our study suggests that BLI can be used as an alternative way to evaluate the efficacy of anti-smallpox treatments. Only in Rag2-/- rats, tissue sections with luminescent signals detected by BLI method showed pathological findings, suggesting that the tissue injuries were specifically induced by vaccinia virus infection, and the immunodeficient rats were more sensitive to vaccinia virus infection than the immunocompetent rats. To further confirm the correlation between bioluminescence signals, pathological injury and vaccinia virus infection, we will perform the immunohistochemical staining for vaccinia virus once good staining antibody were obtained.

VARV infection generally occurs via the respiratory tract and transit to lymph nodes, but infected clothing or bedding also leads to a broad infection, where it replicates and results in a primary viremia in bone marrow, spleen, liver and other organs. Subsequent replication is followed by a secondary viremia, which causes the onset of the symptom, and then the virus seeds next replication targets including the skin on the face and extremities^25^. In the present study, at day 1 post infection of rTV-Fluc, the bioluminescence imaging of SD and Rag2-/- rats could be detected in limbs muscle, skin, ovary and brain. These phenomenon is consistent with previous studies, such as, monkeypox antigen identified in ovarian tissues^26^, and in brain after VACV infection in IFN I R-/- mouse^18^. The viral spreading pattern in SD or Rag2-/- rats was affected by inoculation routes. Both ID and IV inoculation induced initial infection at inoculation sites. The IV injection is not physiological for VARC virus infection, because it bypasses the early stage of smallpox infection via respiratory tract. However, we observed the initial transmission and infection in the primary target tissues and viremia, an outcome of VACV infection in humans. Compared with ID inoculation, IV inoculation resulted in a broader dissemination with the bioluminescence imaging almost in the entire body. Therefore, IV inoculation could be used to test the anti-viral effects of drugs or vaccines in a more challenged condition. Intravenous and intradermal infections were used in this study to simulate the skin contact and secondary viremia respectively. The intranasal infection was also examined for comparison. The dissemination of vaccinia virus infection in Rag2-/- rats was observed from the intranasal inoculation site (day1) to throat (day3), and bioluminescent signal was not detected in the other organs. Meanwhile, the bioluminescent signals were only observed in the inoculation site of SD rats, and no dissemination was detected (Supplementary Fig. 2). Moreover, the repeatability between different rats was poor, and only 33% of Rag2-deficient rats was positive for infections and bioluminescent signals. Therefore, the intranasal route didn’t product consistent results as the other two inoculation routes.

To develop a more effective smallpox vaccine, it is essential to understand the host protection mechanism of the immune system. In our study, the immunodeficient Rag2-/- rats did not develop enlarged primary lesions and distal virus distribution after ID inoculation compared to immunocompetent rats, indicating that the innate immunity of natural killer (NK) cells was critical in controlling the initial VACV infection. Similarly, people with defects in cell-mediated immunity may have an innate antiviral response against vaccinia in their skin^27^. In small animals and humans, prior to the development of robust IgG and cytotoxic lymphocyte (CTLs) responses, neutralizing IgM could control viral infection as early as 4 days after the VACV immunization was given^28^,^29^. In our studies, the neutralizing antibody induced from the rTV-Fluc infection in the immunocompetent but not immunodeficient rats correlated well with the bioluminescent peak signals and the protection level against VACV reinfection, suggesting that B cell function is important for viral defenses. Vijay P *et al.* also showed that protective immunity against secondary poxvirus infection did not require CD4 or CD8 T cell functions but correlated strongly with the generation of neutralizing antibodies^30^. In contrast to our results, others indicated that the recovery from secondary VACV infection in mice did not require B-cell function. Mice deficient in B cells, CD4 cells, or MHC II were fully protected against lethal VACV challenge after they were vaccinated with a highly attenuated vaccinia Ankara strain^31^,^32^. In the absence of antibodies, CTLs were also implicated to contribute to the protective effect against VACV infection by perforin-dependent CD4^+^ T-cell killing^33^ and the antiviral function of type I interferon^18^. This controversy may be partially explained by the diversity of experimental animal models used to explore the relative importance of innate and adaptive immune responses.

Recently, researchers found progressive vaccinia (PV) in VACV-scarified SCID mice, and combination treatment of vaccinia immune globulin and topical cidofovir resulted in a long-term disease-free survival of most of the animals^34^. Similarly, we found that PV could also be induced in immunodeficient Rag2-/- mouse infected with rTV-Fluc via ID inoculation (data not shown). The intensity and duration of bioluminescence signal were enhanced in Rag2-/- rats compared to that in wild type rats, confirming that immunodeficient animals are more susceptible to rTV-Fluc infection. Therefore, immunodeficient Rag2-/- rats are valuable animal models for studying the VACV infection in immunodeficient human populations, and examining the safety and protective efficacy of smallpox vaccines and antiviral drugs. Notably, the reduced pathogenicity of rTV-Fluc in SD may be related to the decreased virulence of recombinant vaccinia virus by insertional knockout of thymidine kinase (TK) gene for rTV-Fluc construction^35^. In the future study, other locus of vaccinia genome which is not related to viral replication and pathogenicity should be chosen, such as *Hind*III F region^36^, for further evaluate the efficacy of orthopoxvirus antiviral drugs.

One animal model never can recapitulate all aspects of human OPV infections, since each model has its own advantages and disadvantages. Visualizing the rTV-Fluc infection in immunocompetent or immunodeficient rats will be an additional or alternative way to study OPV infection, because of its convenient manipulation, sensitivity and small demands of animals. Overall, the rTV-Fluc infected SD or Rag2-/- rats can serve as small animal models to evaluate immunological protection mechanisms and the efficacy of antiviral drugs. Moreover, these models could be used for safety evaluation of the second-generation smallpox vaccine that will be used in humans, and provide further insights into the pathogenesis and treatment of human smallpox.

## Materials and Methods

### Cells and virus

Vero (ATCC, CCL81) cells and 143TK (CCTCC, GDC076) cells were maintained in Dulbecco’s modified Eagle’s medium (DMEM, Hyclone) supplemented with 10% heat-inactivated fetal bovine serum (FBS, Hyclone), 1% L-glutamine and 0.5% combined antibiotics (HyClone, South Logan, UT, USA). Primary chicken embryo fibroblasts (CEF) prepared from 9-day-old embryos were grown in DMEM supplemented with 10% FBS. Vaccinia virus tiantan strain (VTT) was kept in our laboratory.

### Construction of rTV expressing luciferase

The rTV-Fluc was constructed by a standard homologous recombination approach^16^. In brief, the firefly luciferase gene of *Photinus pyralis* was from pLUCF (kindly provided by John T. Schiller, National Cancer Institute, Bethesda, MD, USA) and inserted into the SalI-SmaI site of pSC65 transfer vector (kindly provided by B.Moss, NIAID, NIH, USA).

Moss, NIAID, NIH, USA), under the control of the VACV-specific early-late promoter, and adjacent to the gene that encodes *lacZ* expressed as a screening marker for recombination, which was regulated by the VACV P7.5 late promoter. Primers with restriction enzyme sites of SalI or SmaI used for this experiment were: 5'-GTCGACGCCACCATGGAAGATGCCAAAAA C-3' (sense, SalI site underlined) and 5'-CCCGGGTTACACGGCGATCTTGCCG CCC-3'(antisense, SmaI site underlined). The amplification cycles were 94 °C for 2 min followed by 35 cycles of 94 °C for 30 s, 55 °C for 30 s and 72 °C for 2 min plus an extension of 72 °C for 10 min. Amplified PCR products were purified using a QIAquick PCR purification kit (QIAGEN, Valencia, CA, USA) and were subjected to direct DNA sequencing using an automated ABI 377 DNA sequencer (Applied Biosystems, Foster City, CA, USA). CEF cells were infected with VTT at multiplicity of infection (MOI) of 0.01 and subsequent transfected with the shuttle vector, pSCFluc, designed to recombine specifically with the TK gene of the VTT to obtain rTV-Fluc recombinant viruses, which were selected by blue/white plaque screening. The rTV-Fluc was propagated in 143TK cells, and titrated in Vero cells, as previously described^37^.

### Generation of TALEN-mediated Rag2-/- rat

A total of 4 TALEN pairs were designed against exon 3 of rat Rag2 gene by Beijing Biocytogen Co. Ltd., using the TALEN GoldenGate system. Rag2-specific TALEN plasmids were generated and transfected into rat C6 cells (ATCC, CCL107). Western blotting analysis was performed to confirm TALEN expression and MSDase assay was carried out to examine the TALEN activity. TALEN #38 was chosen for injection into rat zygotes. *In vitro*-transcribed Rag2-TALEN mRNAs were prepared and microinjected into fertilized eggs of SD rats, which were transferred to pseudopregnant females. All rats were maintained in a specific pathogen-free facility. Newborn pups were genotyped by PCR using tail-derived DNAs with specific primers against rat Rag2 as follows: Rag2-F: CCTCGGATTCTCAAAGCAAGGG and Rag2-R: CAGGACGTATGTTACTGGCAAGTG. PCR products were sequenced for Rag2 mutations by Sanger sequencing.

### Flow Cytometry

To detect T, B and NK cells in four-week old Rag2 WT and KO rat, multicolor cytometric analysis was performed using BD FACScalibur (Becton Dickinson, Franklin Lakes, NJ), according to the manufacturer’s protocol. Peripheral blood was taken from the right ventricle of the heart. The spleen cells were collected from the spleen and filtered by the BD cell strainer. The erythrocytes in the blood and spleen cells were depleted by Lysing solution (BD PharMingen, San Diego, CA). White blood cells were examined by single staining or double staining with antibodies including APC-CD3, FITC-CD4, FITC-CD8, FITC-CD45RA, and FITC-CD161a. All antibodies were purchased from BD PharMingen.

### Animal experiments

Rats were housed and handled in accordance with the guidelines set by the Association for the Assessment and Accreditation of Laboratory Animal Care. The study protocol was approved by the NIFDC Institutional Animal Care and Use Committee. Four-week-old female Sprague-Dawley (SD) and Rag2-/- rats were obtained from Institute for Laboratory Animal Resources, NIFDC (Beijing, China). Two groups (3 rats per group) of rats received a single dose of rTV-Fluc at 1 × 10^7^ pfu (suspended in 100 μl of PBS) by intradermal injection on dorsal spine or intravenous injection at 2 × 10^7^ pfu (suspended in 100 μl of PBS). Rats were examined by bioluminescence imaging at 6 h, 1d, 3d, 6d, 9d, 20d and 35d post infection. A second inoculation was performed at 35 days after the first injection via the intradermal injection for all rats, and there are 4 additional SD rats as negative controls. Rats were examined for bioluminescence and antibody titers. To test the pathogenicity of rTV-Fluc in rats, body weight were monitored and recorded. Rats were humanely euthanized with isoflurane for biodistribution analysis of rTV-Fluc in tissues at indicated time points.

### Bioluminescence imaging analysis

Bioluminescence was analyzed using IVIS-Lumina III imaging system (Xenogen, Baltimore, MD) at indicated time points. Acquisition of images and analyzing methods were described previously^19^. In brief, rats were immobilized by an intraperitoneal (i.p.) injection of somnopentyl (240 mg/kg body weight), followed by an i.p. injection of the substrate D-luciferin (50 mg/kg body weight, Xenogen-Caliper Corp., Alameda, CA). Ten minutes later, rats were placed in the imaging chamber of Xenogen *in vivo* imaging system. Rats were positioned to image ventral or dorsal surfaces, and the acquisition time was set at 1 min. For imaging of individual organs, rats were dissected rapidly and imaged *ex vivo* within 3 min to quantify bioluminescence using the IVIS system. The relative intensities of the emitted luminescent light were displayed as pseudocolored images, with colors ranging from red (the most intensive) to blue (the least intensive). Signals emitted from different regions of interest (ROI) of the body were measured and presented as total flux in photons/sec. All data were presented as mean values ± SEM.

### Quantitative PCR analysis of vDNA

vDNA of rTV-Fluc was extracted from different tissues with QIAamp DNA Mini Kit (Qiagen). Primer pairs and probes complementary to the gene segment of the vaccinia tiantan strain and GAPDH are as follows: TA27L (5'-CGTGATCGGTATCTTTCTAG-3', 5'-CCACTACTAATTGGTTCAGTA-3', 5'-FAM-TCTTACCTCTTCTTCAGATCCACACGA-BHQ1-3'); GAPDH (5'-GCTCTCAATGACAACTTTG-3', 5'-GTCCAGGGTTTCTTACTC-3', 5'-FAM-ATGACAATGAATAYGGCTACAGCAAC-BHQ1-3'). qPCR was performed using LightCycler 480 Probes Master Kit (Roche), and presented as vDNA copies/GAPDH ratio.

### Firefly luciferase-based VACV neutralization assay

Neutralization was measured by serum vaccinia NAb induced reduction of rTV-Fluc reporter gene expression^16^. In brief, serial dilutions (starting from 1:30) of sampled sera were mixed with 200 pfu of rTV-Fluc virus in 96-well Costar plates (Corning, Inc., Corning, NY) at 37 °C for 1 h. Then Vero cells were seeded at 3 × 10^4^ cells/well into plates and incubated at 37 °C for 24 h. Sera from naive rats were treated under the same conditions and used as negative controls. After incubation, 100 μL of supernatant was aspirated and 100 μL of D-luciferin substrate (Caliper, Hopkinton, MA, USA) was added into each well at room temperature for 2 min protecting from light. Then the luminescence was measured using a GLOMAX 96 microplate luminometer (Promega, Madison, WI, USA). The 50% neutralization titer (NT50) was defined as the reduction of the relative light unit (RLU) by 50% in serum dilution compared with that in virus-containing controls after subtracting the RLU background in cell-containing controls.

### Histopathology

Several tissues, including lung, heart, spleen, liver, kidney, intestine, skin, muscle, and brain, were obtained from dissected Rag2-deficient and SD rats at day 35 post infection, placed in 10% formalin and then embedded. In Rag2-deficient rats, only regions with strong luminescent intensity were incised under the monitoring of BLI method. The 2μm-thick paraffin sections were stained with haematoxylin and eosin (HE) and observed for pathological changes under light microscope.

### Statistical Analysis

All graphs were generated using Prism 5.0 software (GraphPad, San Diego, CA). Comparisons between the vaccinia neutralizing antibody titers and the bioluminescence were evaluated using Chisquare test. Paired *t* tests were used in other analysis. P values below 0.05 were considered statistically significant.

## Additional Information

**How to cite this article**: Liu, Q. *et al.* Bioluminescent imaging of vaccinia virus infection in immunocompetent and immunodeficient rats as a model for human smallpox. *Sci. Rep.* **5**, 11397; doi: 10.1038/srep11397 (2015).

## Supplementary Material

Supplementary Information
