# Supplementary material for: Bioluminescent imaging of vaccinia virus infection in immunocompetent and immunodeficient rats as a model for human smallpox
Source: Sci Rep. 2015 Aug 3;5:11397. doi: 10.1038/srep11397 (PMC4522659; doi:10.1038/srep11397)
Supplement: Supplementary Information [file srep11397-s1.pdf]

Bioluminescent imaging of vaccinia virus infection in immunocompetent and immunodeficient rats as a model for human smallpox

Qiang Liu[1], Changfa Fan[2], Shuya Zhou[2], Yanan Guo[3], Qin Zuo[2], Jian Ma[1], Susu Liu[2], Xi Wu[2], Zexu Peng[2], Tao Fan[2], Chaoshe Guo[3],Yuelei Shen[3], Weijin Huang[1], Baowen Li[2], Zhengming He[2], and Youchun Wang[1]*

[1]Division of HIV/AIDS and Sex-transmitted Virus Vaccines, Key Laboratory of the Ministry of Health for Research on Quality and Standardization of Biotech Products, National Institutes for Food and Drug Control, Beijing, 100050, China

[2]Division of Animal Model Research, Institute for Laboratory Animal Resources, National Institutes for Food and Drug Control, Beijing, 100050, China

[3]Biocytogen Co., Ltd, Beijing, 101111, China

Q. Liu, C. Fan and S. Zhou contributed equally to this work.

*Correspondence: Youchun Wang, M.D., Ph.D., National Institutes for Food and Drug Control, No. 2 Tiantanxili, Beijing 100050, China. Tel.: +86 10 67095921; Fax: +86 10 67095795. E-mail: wangyc@nifdc.org.cn

Running title: Visualizing vaccinia virus infection in rats

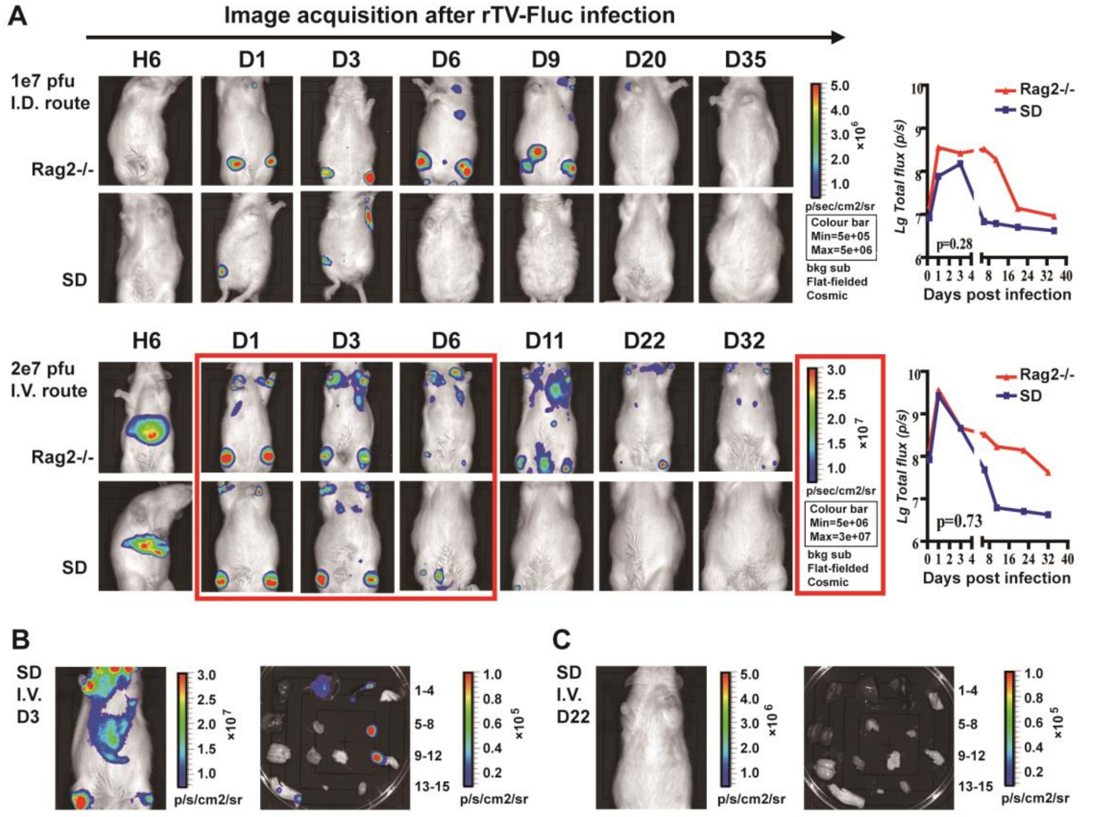

Supplementary Figure 1. Bioluminescence imaging of immunocompetent SD and immunodeficient Rag2-/- rats infected with rTV-Fluc. (A) Comparison of the intensity and duration of ventral bioluminescence between SD rats and Rag2-/- rats inoculated with rTV-Fluc by intradermal (1×10^7 pfu) and intravenous injection (2×10^7 pfu), respectively. Bioluminescent images were superimposed on gray-scale photographs of rats at 6h, 1d, 3d, 6d, 9d, 20d, 35d post intradermal infection and at 6h, 1d, 3d, 6d, 11d, 22d, and 32d post intravenous infection. A representative animal from each group was shown. The relative level of bioluminescence was shown in pseudocolor, with red and blue representing the strongest and weakest photon fluxes, respectively. Values of total flux for each groups was shown on the right and each data point represents a mean value (n=6). The differences between two subgroups were calculated using paired t-test. The living SD rat intravenously

infected with 2×10^7 pfu rTV-Fluc was imaged at day 3 (B) and day 22 (C) after infection. The various organs and tissues were dissected and imaged. 1) heart; 2) liver; 3) spleen; 4) lung; 5) kidney; 6) large intestine; 7) small intestine; 8)ovary; 9) brain; 10) muscle; 11) skin; 12) forelimb; 13) hindlimb; 14) lymph node; 15) mouth.

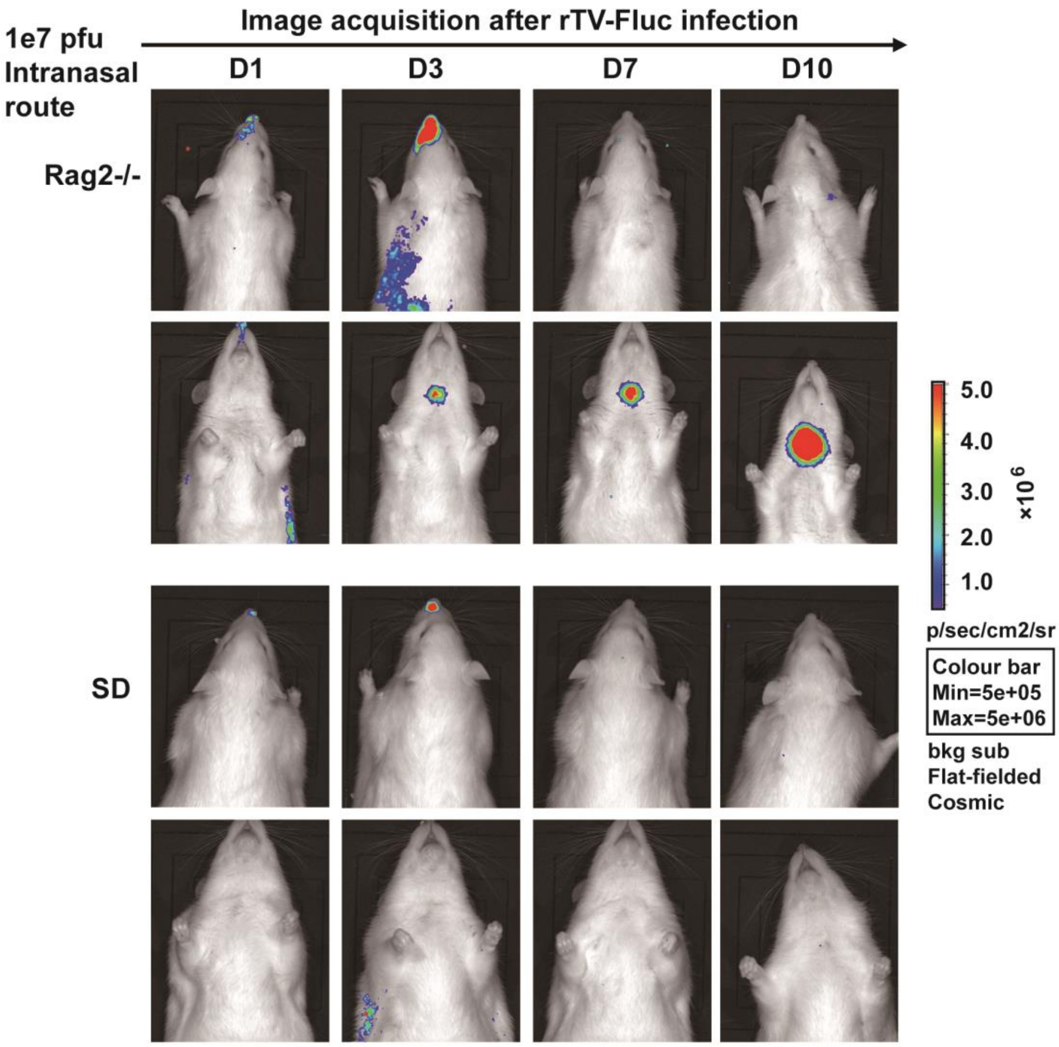

Supplementary Figure 2. The replication and dissemination of rTV-Fluc in Rag2-/- and SD rats. Three 4-week old female Rag2-/- and SD rats were inoculated with rTV-Fluc by intranasal route (1×10^7 pfu in 100ul). The relative level of bioluminescence was shown in pseudocolor, with red and blue representing the strongest and weakest photon fluxes, respectively.
